# Pandemic-Associated Dental Office Closures Associated With Increased Use of Emergency Departments for Dental Conditions in Publicly Insured Children In New York State

**DOI:** 10.1016/j.acepjo.2025.100094

**Published:** 2025-03-11

**Authors:** Shulamite Sian Huang, Scarlett Wang, Heather T. Gold

**Affiliations:** 1 Department of Epidemiology and Health Promotion, New York University College of Dentistry, New York, New York, USA; 2 New York University Wagner Graduate School of Public Service, New York, New York, USA; 3 Department of Population Health, New York University Grossman School of Medicine, New York, New York, USA

**Keywords:** dental, pediatric, pandemic, oral health, dentists

## Abstract

**Objectives:**

All traditional dental clinics were closed from March to May 2020 due to the COVID-19 shutdown, potentially causing additional strain on hospital emergency departments (EDs) to care for patients with dental conditions. We evaluated the impact of pandemic-associated dental office closures on the share of dental conditions managed in EDs among children on Medicaid.

**Methods:**

We quantified the change in the dental-related ED burden among publicly insured children before, during, and after pandemic dental office closures across NY using 2018-2020 New York State (NY) Medicaid claims data among children under age 19 using a difference-in-differences approach.

**Results:**

After controlling for seasonality, dental practice closures in 2020 in NY led to a 2.31 percentage point increase in the share of dental conditions seen in EDs (*P* < .01) among children on Medicaid, representing a 62% increase over 2019 levels. This was sustained even after reopening in May 2020 (1.26 percentage point increase in the reopening phase, *P* < .01). The increases in the dental-related ED burden during dental office closures were due to the increased use of EDs for dental conditions.

**Conclusion:**

Lack of access to dental care during a time of significant health care system strain was associated with an increased burden on EDs from dental conditions among publicly insured children. Health care systems should consider alternatives to referral programs to dental offices to ensure publicly insured children do not fall through the dental safety net, such as by providing limited dental services on-site or incorporating urgent dental care clinics within hospitals.

**The Bottom Line**

Dental office closures during the pandemic were associated with a 62% increase in the burden on emergency departments (EDs) from dental conditions among Medicaid-enrolled children, which was sustained even after dental offices reopened in May 2020. The increased use of EDs for dental conditions may reflect an increased strain on health care systems during a time of limited health care resources that was preventable.

## 1 Introduction

### 1.1 Background

Though dental complaints account for only 1% to 2% of annual emergency department (ED) visits in the United States,^1^ pandemic dental office closures may have increased substitution toward EDs for dental conditions. Though individuals with dental emergencies could still access dental care under social distancing guidelines,^2^, ^3^, ^4^ it is unclear to what extent dental care access was available, especially for populations with limited access to dental care services. The Medicaid pediatric population is one such population that faces limited dental care access—though many states provide comprehensive pediatric Medicaid dental coverage, dentist participation in Medicaid is limited^5^, ^6^, ^7^ and highly dependent on reimbursement rates.^6^^,^^7^ Additionally, the emergency declaration limited access to other sources of dental care for low-income children, such as school-based caries prevention programs. Hence, the available sources of dental services for Medicaid-insured children may have diminished significantly upon the onset of the COVID-19 pandemic.

### 1.2 Importance

Without access to usual sources of dental care, parents are more likely to choose EDs. However, prior work has emphasized that EDs are not well equipped to treat dental conditions, are limited generally to providing palliative care and pain relief, and require a referral to a dental office to address the underlying issue.^1^^,^^8^, ^9^, ^10^ Hence, ED visits for dental conditions represent a waste of hospital and health care system resources.^1^^,^^8^, ^9^, ^10^ The potential decline in dental care access due to the pandemic may have inadvertently increased the burden on general health care systems for dental conditions among children.

Recent work using data from a single pediatric hospital has suggested dental office closures lead to an increase in the proportion of dental-related ED concerns among children;^11^ however, it is unknown to what extent this phenomenon was experienced across health care systems. Similarly, though recent work has demonstrated declines in both overall ED and dental-related ED visits during the pandemic,^12^^,^^13^ the extent to which the location of care for dental needs shifted toward EDs and away from dental offices during the pandemic is unknown. We focus on the New York State (NY) Medicaid pediatric population, because both comprehensive dental and medical benefits are provided across this population, and the resulting claims data provide a comprehensive view of how trends in dental and dental-related medical care utilization may have changed across multiple venues of care (ie, dental offices, outpatient medical clinics, EDs, and teledentistry).

### 1.3 Goals of This Investigation

The aim of this study is, therefore, to assess the following: (1) the impact of dental office closures during the pandemic on the dental-related ED burden and on dental and dental-related medical visits in a low-income pediatric population across an entire state encompassing multiple health care systems; (2) whether the dental office closures were associated with changes in the location of care for dental needs, and whether such changes were sustained even after dental offices reopened; and (3) whether the pandemic impact varied by age group and geographic characteristics.

## 2 Methods

### 2.1 Study Design, Selection of Subjects, and Setting

In this retrospective observational study, we used claims data from the New York Medicaid program between January 1, 2018, to December 31, 2020, consisting of New York Medicaid enrollees under age 19 years, totaling 2.46 million pediatric beneficiaries in 2020, 2.42 million beneficiaries in 2019, and 2.38 million beneficiaries in 2018. The insurance claims data include comprehensive medical and dental claims for all enrollees, as well as information on the enrollee date of birth and zip code of residence. We supplemented the claims data with county sociodemographic variables from the 2018 American Community Survey,^14^ the 2013 urban-rural classification for counties from the National Center for Health Statistics,^15^ and county dental health professional shortage area (DPSA) status from the Health Resources and Services Administration.^16^ This study was granted an exemption by the New York University School of Medicine Institutional Review Board and follows the Strengthening the Reporting of Observational Studies in Epidemiology (STROBE) reporting guideline.

### 2.2 Measures/Outcomes

Our primary measure of interest is the dental-related ED burden, quantified by the share of ED visits resulting from nontraumatic dental conditions (NTDC), along with key components influencing the NTDC ED burden. These key components were derived using the following arithmetic identity, similar to prior work^1^:

$$\frac{\#\;NTDC\;ED\;visits}{\#\;ED\;visits}=\frac{\#\;NTDC\;ED\;visits}{\#\;dental-related\;visits}\;x\;\frac{\#\;dental-related\;visits}{\#\;population}x\;\frac{\#\;popn}{\#\;ED\;visits}$$

The NTDC ED burden is, therefore, a function of the following: (1) the proportion of dental-related visits that occur in EDs, (2) the rate of dental-related medical and dental care utilization in the population, and (3) the rate of ED utilization in the population. Increased NTDC ED burden could result from increased use of EDs for dental conditions, increased dental-related care utilization rate, or decreased ED utilization rate.

We identified NTDC ED visits using a set of ICD-10 codes to identify NTDCs according to the latest recommendations from state dental directors.^17^^,^^18^ Specifically, this excludes ED visits resulting from trauma and includes visits for conditions such as dental caries, periapical abscesses, and periodontitis.^19^ A complete list of the ICD-10 codes used is available in the prior literature.^17^^,^^18^^,^^20^ We then quantified the NTDC burden in EDs among children with NY Medicaid as the proportion of ED visits with an NTDC diagnosis.

To quantify the dental-related medical and dental care utilization rates per 10,000 NY Medicaid beneficiaries, we identified both dental and dental-related medical care visits within the NY Medicaid claims. Dental visits include in-person dental clinic visits, in-person school-based dental visits, and teledentistry visits. Dental-related medical visits include in-person medical outpatient visits with dental procedure codes^21^ or NTDC ED visits.^20^ In-person dental visits were identified through claims billed by dentists. To ensure that we include other sources of dental care for Medicaid beneficiaries outside of ED visits and dental visits, we identified in-person dental-related outpatient visits by identifying physicians or nurse practitioners billing with dental procedure codes (CDT) in non-ED outpatient settings (non-ED outpatient settings are identified using a category of service variable, which identifies the venue of service and distinguishes between ED and non-ED outpatient venues). School-based dental care was then identified by school-based health programs billing both CDT codes and school health-specific rate codes. We identified teledentistry services by using CDT codes D9995 and D9996.^22^

We quantified the all-cause ED utilization rate per 10,000 beneficiaries. Multiple claims for ED visits occurring within the same day for the same beneficiary were assumed to be for the same visit. The percentage of dental-related medical and dental visits occurring within EDs was then calculated by dividing the number of NTDC ED visits in a county for each week by the total number of dental-related medical and dental visits occurring within a county in each week.

### 2.3 Data Analysis

We estimated the impact of dental office closures and reopenings on the NTDC ED burden by using an age group level difference-in-differences (DID) model that compared the change in the proportion of ED visits in weeks 11 to 21 (weeks of dental office closure) and 22 to 52 (weeks of dental office reopenings) versus weeks 1 to 10 in 2020 with the change in the same period in 2018 and 2019. Week and year fixed effects were additionally included to capture potential seasonal trends. We then estimated the impact of dental office closures and reopening on the dental care utilization rate and components of the NTDC ED burden by using a county-level DID model estimated with generalized least squares^23^, ^24^, ^25^ with a log link, Poisson distribution, and robust standard errors. We followed Shang et al (2018)^25^ to convert the coefficients of interest *θ* and *μ* into difference-in-semielasticities (DIS), ie, the percentage change in the dependent variable in response to a single unit change in the independent variable.^25^ We then quantified the amount of change in the NTDC burden in ED visits attributable to the change in each individual component from the dental office closure policy, assuming other components are held fixed at their prepandemic levels. Finally, we accounted for heterogeneity across age groups and counties by stratifying analyses by age group and county characteristics. Analyses were not adjusted for multiple comparisons. Data cleaning, processing, and summary statistics were conducted using SAS version 9.4. Analyses were conducted using Stata version 16.1.

## 3 Results

Table 1 describes the enrollees under age 19 years in the NY Medicaid claims data by sociodemographic characteristics by year. The total number of enrollees declined from 2018 to 2019 and 2020, from 2.46 million enrollees to 2.36 million enrollees under age 19. There were no substantial changes in the demographic composition between 2018 through 2020, whereas the percentage of enrollees having any dental or dental-related medical care utilization during the year declined in 2020 from prior years.

**Table 1:** Summary statistics of enrollees in each year.

Demographic characteristics	Year		
	2018	2019	2020
Total unique enrollees (N)	2,460,772	2,426,819	2,368,150
Percent male	50.9%	50.9%	51.0%
Mean age (SD)	9.32 (5.95)	9.45 (5.94)	9.51 (5.91)
Percent in the age group			
0-4 y	28.2%	27.6%	26.8%
5-9 y	25.1%	25.0%	25.0%
10-14 y	23.9%	24.3%	24.8%
15-19 y	22.8%	23.1%	23.4%
Percent below poverty line			
1st quartile (lowest)	17.9%	18.0%	18.2%
2nd quartile	6.1%	6.2%	6.3%
3rd quartile	32.6%	32.7%	32.9%
4th quartile (highest)	43.4%	43.1%	42.7%
Percent residing in metropolitan areas	10.0%	10.0%	9.8%
Percent any dental or dental-related medical utilization during the year	45.2%	47.0%	36.1%
Dental and dental-related medical care utilization per 10K pediatric Medicaid enrollees			
Mean weekly # dental office visit rate (SD)	225.6 (63.4)	224.1 (60.1)	153.6 (88.7)
Mean weekly # dental ED visit rate (SD)	3.6 (3.0)	3.6 (2.9)	2.5 (2.4)
Mean weekly # all-cause ED visit rate	92.3 (33.7)	95.1 (32.9)	57.4 (34.4)

ED, emergency department.

Visit rates are calculated per 10K pediatric Medicaid enrollees.

Figure maps the trend in NTDC ED admissions as a percentage of total ED admissions by age group and Table 2 summarizes the impact across the COVID shutdown period using DID analysis at the age group-week-year level. We find that across the pediatric Medicaid population, there is an increase of 2.3 percentage points in the share of ED visits from NTDCs from dental office closures in 2020 relative to the same weeks in prior years. The increase is concentrated among very young children, primarily between the ages of 0 to 4 and 5 to 9 years. Children between the ages of 0 to 4 and 5 to 9 years experience nearly a 2-fold increase in the proportion of ED visits from NTDCs during the weeks of dental office closures in 2020 compared to the same weeks in 2019 (Fig and Table 2). In contrast, children aged 10 to 14 years and those aged 15 to 19 years faced more muted increases in the NTDC ED burden during dental office closures, with the increase among ages 15 to 19 years being statistically insignificant.

**Figure:**
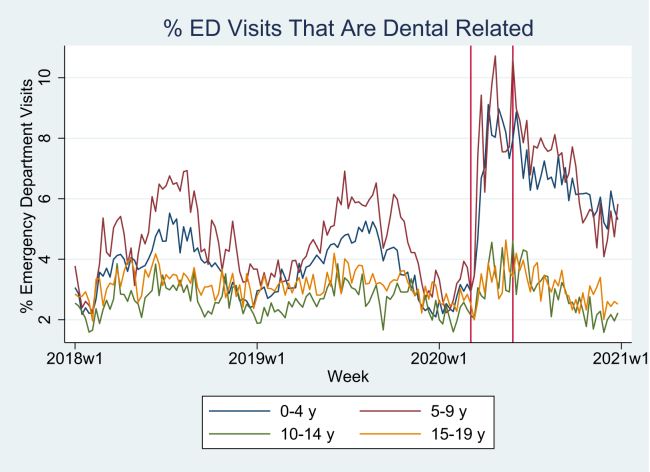
The trend in dental-related emergency department (ED) admissions as a percentage of total ED admissions, by age group.

**Table 2:** Regression results for NTDC ED burden (at the age group-week-year level).

	(1)	(2)	(3)	(4)	(5)
Dependent variable: percent of ED visits that are dental-related					
	All ages (0 to 19 y)	0 to 4 y	5 to 9 y	10 to 14 y	15 to 19 y
Closure × 1 (2020)	2.307^a^	3.748^a^	4.004^a^	0.704^b^	0.325
	(0.36)	(0.46)	(0.61)	(0.27)	(0.24)
Reopening × 1 (2020)	1.258^a^	2.706^a^	1.917^a^	–0.029	0.083
	(0.31)	(0.39)	(0.52)	(0.23)	(0.20)
Year FE	Yes	Yes	Yes	Yes	Yes
Week FE	Yes	Yes	Yes	Yes	Yes
Observations	156	156	156	156	156
R-squared	0.601	0.736	0.597	0.265	0.146
Mean percent dental ED in weeks 11-52 in 2019	3.721	3.803	4.709	2.770	3.235

ED, emergency department; FE, fixed effects; NTDC, nontraumatic dental conditions.

This table displays the estimates of the impact of the pandemic dental office closures on the percent of ED visits that are dental-related, along with robust standard errors (in parentheses). Closure × 1 (2020) is equal to 1 for weeks 11 through 21 in 2020, whereas reopening × 1 (2020) is equal to 1 for weeks 22 to 52 in 2020. This was estimated via ordinary least squares with robust standard errors. Observations are at the age group-week-year level.

a *P* < .01.

b *P* < .05.

Table 3 demonstrates that dental office closures were associated with the largest and most lasting declines in dental office utilization among children aged 0 to 4 years. Few dental office visits took place among children aged 0 to 4 years during dental office closures, with a 99% decrease in dental office visits during weeks of dental office closures in 2020 relative to the same weeks in 2018 and 2019 (Table 3, *P* < .01). In contrast, children in progressively older age groups saw progressively smaller declines in dental office utilization during dental office closures, whereby children aged 5 to 9, 10 to 14, and 15 to 19 years saw declines of 92.5%, 83.0%, and 81.3%, respectively (Table 3, all *P* < .01). Similarly, children aged 0 to 4 years sustained the largest declines in dental office visit rates after dental offices reopened relative to the same weeks in 2018 and 2019 (31.2% decline, *P* < .01).

**Table 3:** The effect of dental office closures and reopenings on key components of NTDC burden in EDs, Poisson regression, DID estimates, and standard errors calculated using Shang et al.^25^

	Components			
	(1)	(2)	(3)	(4)
	All-cause ED visit rate	All dental and dental-related visit rate	Proportion of dental in ED	Dental office visit rate
Panel A: ages 0-4 y				
Closure × 1 (2020)	–0.814^a^^,^^b^^,^^c^	–0.855^a^^,^^b^^,^^c^	1.603^a^^,^^b^^,^^c^	–0.986^a^^,^^b^^,^^c^
	(0.0351)	(0.0205)	(0.119)	(0.0185)
Reopening × 1 (2020)	–0.653^a^^,^^b^^,^^c^	–0.288^a^^,^^b^^,^^c^	0.03840	–0.312^a^^,^^b^^,^^c^
	(0.0313)	(0.0298)	(0.0409)	(0.0328)
County FE	Yes	Yes	Yes	Yes
Year FE	Yes	Yes	Yes	Yes
Observations	9672	9672	9548	9672
Prepandemic dep var mean	131.1	155.9	0.0383	114.3
During pandemic dep var mean	55.36	97.56	0.0560	66.71
Panel B: ages 5-9 y				
Closure × 1 (2020)	–0.955^a^^,^^b^^,^^c^	–0.985^a^^,^^b^^,^^c^	2.906^a^^,^^b^^,^^c^	–0.925^a^^,^^b^^,^^c^
	(0.0459)	(0.0253)	(0.357)	(0.0151)
Reopening × 1 (2020)	–0.675^a^^,^^b^^,^^c^	–0.334^a^^,^^b^^,^^c^	–0.0386	–0.252^a^^,^^b^^,^^c^
	(0.0416)	(0.0382)	(0.0413)	(0.0293)
County FE	Yes	Yes	Yes	Yes
Year FE	Yes	Yes	Yes	Yes
Observations	9672	9672	9566	9672
Prepandemic dep var mean	72.81	312.0	0.0123	272.0
During pandemic dep var mean	31.60	178.6	0.0287	157.7
Panel C: ages 10-14 y				
Closure × 1 (2020)	–0.915^a^^,^^b^^,^^c^	–0.857^a^^,^^b^^,^^c^	0.876^a^^,^^b^^,^^c^	–0.830^a^^,^^b^^,^^c^
	(0.0437)	(0.0130)	(0.202)	(0.0134)
Reopening × 1 (2020)	–0.512^a^^,^^b^^,^^c^	–0.271^a^^,^^b^^,^^c^	–0.256^a^^,^^b^^,^^c^	–0.236^a^^,^^b^^,^^c^
	(0.0369)	(0.0257)	(0.0762)	(0.0256)
County FE	Yes	Yes	Yes	Yes
Year FE	Yes	Yes	Yes	Yes
Observations	9672	9672	9488	9672
Prepandemic dep var mean	71.77	308.7	0.00647	285.5
During pandemic dep var mean	36.86	182.5	0.00997	170.4
Panel D: ages 15-19 y				
Closure × 1 (2020)	–0.759^a^^,^^b^^,^^c^	–0.804^a^^,^^b^^,^^c^	1.056^a^^,^^b^^,^^c^	–0.813^a^^,^^b^^,^^c^
	(0.0376)	(0.00845)	(0.168)	(0.00945)
Reopening × 1 (2020)	–0.378^a^^,^^b^^,^^c^	–0.203^a^^,^^b^^,^^c^	–0.111	–0.203^a^^,^^b^^,^^c^
	(0.0290)	(0.0198)	(0.0764)	(0.0208)
County FE	Yes	Yes	Yes	Yes
Year FE	Yes	Yes	Yes	Yes
Observations	9672	9672	9589	9672
Prepandemic dep var mean	102.9	270.5	0.0125	250.2
During pandemic dep var mean	64.44	164.3	0.0180	150.0

DID, difference-in-differences; ED, emergency department; FE, fixed effects; NTDC, nontraumatic dental conditions.

This table displays the difference-in-semielasticities (DIS) estimates of the impact of the pandemic dental office closures on the percent of ED visits that are dental-related, along with robust standard errors for the DIS estimates (in parentheses). Observations are at the age group-week-county level. Closure × 1 (2020) is equal to 1 for weeks 11 through 21 in 2020, whereas reopening × 1 (2020) is equal to 1 for weeks 22 to 52 in 2020.

The decline in observations in column (3) is due to some counties having no ED visits within a specific week. “Dep Var Mean” refers to “Dependent Variable Mean Prior to COVID Pandemic Across Weeks” which is the calculated mean of the dependent variable in each column across all weeks between January 1, 2018, to March 11, 2020. For instance, there was an average of 131.1 all-cause ED visits per week per 10K Medicaid enrollees between the ages of 0 to 4 years prior to the pandemic.

a *P* < .01.

b *P* < .05.

c *P* < .1.

Increased use of EDs for dental services was the primary driver in the change in the NTDC ED burden during the dental office closures (Table 3). During the dental office closures, the decline in the all-cause ED utilization rate was offset by a similarly sized decline in the dental-related service utilization rate. However, among ages 0 to 4 and 5 to 9 years, there are strong shifts toward the ED for dental services with a respective 160% and 291% increase in the proportion of dental visits occurring in the ED in weeks of dental office closures in 2020, relative to the same weeks in 2018 and 2019 (both *P* < .01). Children aged 10 to 14 years and those aged 15 to 19 years exhibited smaller increases in ED use for dental services relative to the younger age groups (Table 3). However, after dental offices reopened, the shifts in the location of care seen during closures were not sustained (Table 3).

After decomposing the NTDC ED burden into its components, we found that dental and dental-related medical care utilization recovered roughly twice as quickly to baseline levels compared to the all-cause ED utilization rate. In the reopening phase, the rate of dental and dental-related care utilization recovered to 28.8% below prepandemic levels in weeks 22 to 52 of prior years for children aged 0 to 4 years (*P* < .01), whereas the rate of all-cause ED utilization remained 65.3% below prepandemic levels (*P* < .01) (Table 3). Similar patterns were seen among those aged 5 to 9 years (Table 3).

Although rural counties faced greater relative declines in dental office visits relative to urban counties (Table S1), there were no clear patterns on how changes in the location of dental- and dental-related medical care associated with dental office closures occurred across rural and urban counties (Table S1). Though children aged 5 to 9 and 15 to 19 years in rural counties saw more dramatic increases in the use of EDs for dental conditions relative to those in urban counties (Table S1), this pattern was reversed among children aged 0 to 5 and 10 to 14 years (Table S1), whereby increases in the use of the ED for dental conditions were greater in urban counties.

Changes in the location of dental- and dental-related care associated with the pandemic dental office closures were sustained even after dental office reopening for 2 groups: (1) children aged 0 to 4 years in rural counties, and (2) those aged 10 to 14 years in urban counties (Table S1). For all other age groups in rural and urban counties, changes in the location of dental and dental-related medical care were not sustained through the reopening period and reverted to prepandemic patterns of utilization.

Counties without dental shortages experienced steeper declines in the dental office utilization rate across all age groups, relative to counties with dental shortages (Table S2). Children aged 0 to 4 years in non-DPSA counties experienced a 100% decline in the number of dental office visits per 10,000 beneficiaries in closure weeks in 2020 relative to the same weeks in prior years (*P* < .01), whereas children of the same age in DPSA counties faced a 94% decline in closure weeks in 2020 relative to the same weeks in prior years (*P* < .01). Similar patterns were found among other age groups across counties with and without dental shortages (Table S2).

Counties without dental shortages also experienced increased use of EDs for dental needs during dental office closures for the 2 youngest age groups, relative to counties with dental shortages (Table S2). For instance, children aged 0 to 4 years in counties without dental shortages experienced a 171% increase in the proportion of dental visits occurring in the ED during weeks 11 to 21 in 2020 relative to the same weeks in prior years (*P* < .01), whereas those in counties with dental shortages experienced a 139% increase (*P* < .01). In contrast, children in the 2 oldest age groups faced more use of EDs for dental needs in counties with dental shortages during office closures relative to counties without dental shortages. Similar patterns were found for children aged 10 to 14 years across counties with and without dental shortages. Changes in the location of dental and dental-related medical care associated with dental office closures in 2020 relative to prior years were not sustained for most age groups in areas with and without dental shortages.

## 4 Limitations

Though the results suggest that low-income children and their families substituted away from dental offices toward EDs for dental conditions during the pandemic-related dental office closures, we are unable to say definitively that this was a substitution, because the NY Medicaid claims data does not record whether patients visiting EDs for dental conditions would have considered dental offices as an option for care.

This study included only children who were enrolled in NY Medicaid and excluded those children who enrolled in NY’s Children’s Health Insurance Program (CHIP), which accounts for 27% of the total pediatric Medicaid/CHIP population. Similarly, we are unable to examine changes in the location of care for children with private health and dental insurance, which requires linkages between claims data across private health insurers and private dental insurers. However, this paper provides a framework with which to examine changes in the utilization patterns in the location of dental and dental-related medical care.

Though parents may have sought care for their children via telehealth services, claims for telehealth services that were not billed by a dentist would have been denied and not captured in the NY Medicaid claims data.^26^ Further work is needed to examine the impact of dental office closures on telehealth visits for dental complaints that were provided by nondentists.

## 5 Discussion

The sizable and statistically significant changes in the NTDC ED burden among children aged 0 to 4 years suggest that the youngest children were more likely to fall through holes in the dental safety net. There are several possible reasons for this. First, parents may have difficulty ascertaining whether young children exhibiting symptoms of pain are experiencing an emergency. Second, though the American Academy of Pediatrics recommends children establish a dental home before the age of 1,^27^ a 2012 study found that in 46 states, <20% of publicly insured children receive their first dental visit before the age of 3 years.^28^ Third, prior literature has highlighted that the majority of general dentists do not see children under the age of 3 years,^29^, ^30^, ^31^, ^32^, ^33^, ^34^ due to lack of training or experience in treating very young children^29^ and low Medicaid reimbursement rates.^29^^,^^35^ Hence, young children exhibiting pain during the pandemic may have been less likely to have had an established dental home and thereby were less able to schedule emergency dental appointments, which dentists were able to continue offering even during pandemic-related closures.^2^, ^3^, ^4^

The subgroup analyses examining DPSAs are counterintuitive because non-DPSA counties faced the following: (1) greater declines in dental care utilization rates, and (2) increased use of EDs for dental needs compared to DPSA counties. This suggests that DPSAs as currently defined may not capture areas of dental shortage for the youngest age groups because DPSA designations are not for the pediatric population, but for the broader population.^36^ The shifts to EDs for dental conditions during dental office closures in non-DPSA counties may indicate a shortage of Medicaid pediatric dental services that is not detected when examining dental care for all populations at the county level. Future work should examine whether levels of dental care access vary by age group and by insurance status.

This is the first paper to examine the impact of widespread declines in dental care access on changes in the location of care for dental conditions for publicly insured children across multiple health care systems. Though recent work has reported on changes in NTDC ED visits^12^ and dental visits^37^^,^^38^ due to the pandemic separately, no work has examined the extent to which the pandemic was associated with changes in the location of services. The increase in the proportion of NTDCs in the ED during and after dental office closures is quantitatively similar to those found in a recent study examining NTDC ED visits in a single medical center with local dental office closures.^11^

In summary, our findings suggest that although dental offices were allowed to see patients and treat dental emergencies, Medicaid-insured children may have had difficulty accessing dental care during the initial phase of the COVID-19 pandemic, leading to an increase in the proportion of ED visits from NTDCs. Health care systems may wish to consider alternatives to ED referral programs to dental offices to ensure publicly insured children do not fall through the dental safety net, such as by providing limited dental services on-site or incorporating urgent dental care clinics within hospitals.

## Author Contributions

S.S.H. conceived the study and obtained research funding. S.S.H. and S.W. analyzed the data, and H.T.G. provided statistical advice on study design. S.S.H. drafted the manuscript, and all authors contributed substantially to its revision. S.S.H. takes responsibility for the paper as a whole.

## Funding and Support

This work is funded by the 10.13039/100000072National Institute of Dental and Craniofacial Research at the 10.13039/100000002National Institutes of Health, under K25 DE028584 and K25 DE028584-02S1.

## Data Sharing Statement

The data use agreement between NYU and the New York State Department of Health does not permit the sharing of person-level data because the administrative data used in this study were initially collected for administration of health care services.

## Disclaimer

The views and opinions expressed in this article are those of the author(s) and do not necessarily reflect the official policy or position of the New York State Department of Health. Examples of analysis performed within this article are only examples. They should not be utilized in real-world analytic products.

## Conflict of Interest

All authors have affirmed they have no conflicts of interest to declare.
