# Supplementary material for: Pandemic-Associated Dental Office Closures Associated With Increased Use of Emergency Departments for Dental Conditions in Publicly Insured Children In New York State
Source: J Am Coll Emerg Physicians Open. 2025 Mar 11;6(2):100094. doi: 10.1016/j.acepjo.2025.100094 (PMC11932651; doi:10.1016/j.acepjo.2025.100094)
Supplement: Supplementary Tables 1-2 [file mmc1.docx]

**Appendix**

Table S1. The effect of dental office closures and reopenings on visit rates for rural vs. urban areas, Poisson regression, DID estimates and standard errors calculated using Shang et al. (2018)

| URBAN COUNTIES | | | | |  | RURAL COUNTIES | | | | |
| --- | --- | --- | --- | --- | --- | --- | --- | --- | --- | --- |
|  | **All-Cause ED Visits** | **All Dental and Dental-Related Visits** | **Proportion Dental in ED** | **Dental Office Visits** |  |  | **All-Cause ED Visits** | **All Dental and Dental-Related Visits** | **Proportion Dental in ED** | **Dental Office Visits** |
| ***Panel A: Ages 0 to 4*** |  |  |  |  |  | ***Panel A: Ages 0 to 4*** |  |  |  |  |
| Closure x 1(2020) | -0.827*** | -0.855*** | 1.643*** | -0.985*** |  | Closure x 1(2020) | -0.632*** | -0.850*** | 1.284*** | -1.007*** |
|  | (0.0347) | (0.0217) | (0.133) | (0.0194) |  |  | (0.0385) | (0.0317) | (0.230) | (0.0319) |
| Reopening x 1(2020) | -0.665*** | -0.289*** | 0.0264 | -0.316*** |  | Reopening x 1(2020) | -0.483*** | -0.263*** | 0.167* | -0.247*** |
|  | (0.0308) | (0.0316) | (0.0426) | (0.0349) |  |  | (0.0377) | (0.0382) | (0.0859) | (0.0408) |
| County FE | X | X | X | X |  | County FE | X | X | X | X |
| Year FE | X | X | X | X |  | Year FE | X | X | X | X |
| N | 5928 | 5928 | 5912 | 5928 |  | N | 3744 | 3744 | 3636 | 3744 |
| Weekly Mean Pre-COVID | 123.1 | 158.8 | 0.0344 | 117.9 |  | Weekly Mean Pre-COVID | 143.7 | 151.3 | 0.0447 | 108.5 |
| ***Panel B: Ages 5 to 9*** |  |  |  |  |  | ***Panel B: Ages 5 to 9*** |  |  |  |  |
| Closure x 1(2020) | -0.973*** | -0.984*** | 2.800*** | -0.923*** |  | Closure x 1(2020) | -0.701*** | -1.003*** | 4.020*** | -0.966*** |
|  | (0.0446) | (0.0267) | (0.372) | (0.0157) |  |  | (0.0545) | (0.0521) | (0.689) | (0.0250) |
| Reopening x 1(2020) | -0.686*** | -0.338*** | -0.0475 | -0.255*** |  | Reopening x 1(2020) | -0.518*** | -0.271*** | 0.0856 | -0.206*** |
|  | (0.0424) | (0.0403) | (0.0429) | (0.0310) |  |  | (0.0422) | (0.0471) | (0.130) | (0.0298) |
| County FE | X | X | X | X |  | County FE | X | X | X | X |
| Year FE | X | X | X | X |  | Year FE | X | X | X | X |
| N | 5928 | 5928 | 5894 | 5928 |  | N | 3744 | 3744 | 3672 | 3744 |
| Weekly Mean Pre-COVID | 68.51 | 322.6 | 0.0117 | 280.9 |  | Weekly Mean Pre-COVID | 79.61 | 295.1 | 0.0132 | 257.9 |

Table S1 cont. The effect of dental office closures and reopenings on visit rates for rural vs. urban areas, Poisson regression, DID estimates and standard errors calculated using Shang et al. (2018)

| URBAN COUNTIES | | | | |  | RURAL COUNTIES | | | | |
| --- | --- | --- | --- | --- | --- | --- | --- | --- | --- | --- |
| ***Panel C: Ages 10 to 14*** | **All-Cause ED** | **All Dental and Dental-Related** | **Proportion of Dental in ED** | **Dental Office** |  | ***Panel C: Ages 10 to 14*** | **All-Cause ED** | **All Dental and Dental-Related** | **Proportion of Dental in ED** | **Dental Office** |
| Closure x 1(2020) | -0.929*** | -0.854*** | 0.884*** | -0.827*** |  | Closure x 1(2020) | -0.751*** | -0.907*** | 0.788* | -0.891*** |
|  | (0.0443) | (0.0136) | (0.219) | (0.0135) |  |  | (0.0394) | (0.0184) | (0.436) | (0.0278) |
| Reopening x 1(2020) | -0.517*** | -0.272*** | -0.268*** | -0.237*** |  | Reopening x 1(2020) | -0.451*** | -0.243*** | -0.130 | -0.219*** |
|  | (0.0394) | (0.0271) | (0.0815) | (0.0269) |  |  | (0.0380) | (0.0266) | (0.170) | (0.0336) |
| County FE | X | X | X | X |  | County FE | X | X | X | X |
| Year FE | X | X | X | X |  | Year FE | X | X | X | X |
| N | 5928 | 5928 | 5918 | 5928 |  | N | 3744 | 3744 | 3570 | 3744 |
| Weekly Mean Pre-COVID | 64.47 | 321.9 | 0.00588 | 297.8 |  | Weekly Mean Pre-COVID | 83.33 | 287.6 | 0.00740 | 265.9 |
| ***Panel D: Ages 15 to 19*** |  |  |  |  |  | ***Panel D: Ages 15 to 19*** |  |  |  |  |
| Closure x 1(2020) | -0.782*** | -0.802*** | 1.010*** | -0.812*** |  | Closure x 1(2020) | -0.549*** | -0.824*** | 1.493*** | -0.829*** |
|  | (0.0361) | (0.00887) | (0.176) | (0.00977) |  |  | (0.0497) | (0.0230) | (0.428) | (0.0303) |
| Reopening x 1(2020) | -0.391*** | -0.201*** | -0.127 | -0.201*** |  | Reopening x 1(2020) | -0.262*** | -0.240*** | 0.0572 | -0.240*** |
|  | (0.0301) | (0.0208) | (0.0805) | (0.0217) |  |  | (0.0432) | (0.0254) | (0.161) | (0.0323) |
| County FE | X | X | X | X |  | County FE | X | X | X | X |
| Year FE | X | X | X | X |  | Year FE | X | X | X | X |
| N | 5928 | 5928 | 5915 | 5928 |  | N | 3744 | 3744 | 3674 | 3744 |
| Weekly Mean Pre-COVID | 91.24 | 273.5 | 0.0112 | 253.4 |  | Weekly Mean Pre-COVID | 121.4 | 265.8 | 0.0145 | 245.0 |

Notes:

Table S2. The effect of dental office closures and reopenings on visit rates for counties with and without dental shortage, Poisson regression, DID estimates and standard errors calculated using Shang et al. (2018)

| COUNTIES WITHOUT DENTAL SHORTAGE | | | | |  | COUNTIES WITH DENTAL SHORTAGE | | | | |
| --- | --- | --- | --- | --- | --- | --- | --- | --- | --- | --- |
|  | **All-Cause ED Visits** | **All Dental and Dental-Related Visits** | **Proportion Dental in ED** | **Dental Office Visits** |  |  | **All-Cause ED Visits** | **All Dental and Dental-Related Visits** | **Proportion Dental in ED** | **Dental Office Visits** |
| ***Panel A: Ages 0 to 4*** |  |  |  |  |  | ***Panel A: Ages 0 to 4*** |  |  |  |  |
| Closure x 1(2020) | -0.827*** | -0.875*** | 1.714*** | -1.004*** |  | Closure x 1(2020) | -0.774*** | -0.803*** | 1.391*** | -0.939*** |
|  | (0.0415) | (0.0150) | (0.137) | (0.0135) |  |  | (0.0706) | (0.0229) | (0.107) | (0.0254) |
| Reopening x 1(2020) | -0.666*** | -0.317*** | 0.0430 | -0.347*** |  | Reopening x 1(2020) | -0.615*** | -0.213*** | 0.03120 | -0.217*** |
|  | (0.0369) | (0.0233) | (0.0488) | (0.0245) |  |  | (0.0633) | (0.0286) | (0.0735) | (0.0299) |
| County FE | X | X | X | X |  | County FE | X | X | X | X |
| Year FE | X | X | X | X |  | Year FE | X | X | X | X |
| N | 5772 | 5772 | 5680 | 5772 | N | N | 3900 | 3900 | 3868 | 3900 |
| Weekly Mean Pre-COVID | 126.6 | 162.7 | 0.0355 | 119.2 |  | Weekly Mean Pre-COVID | 137.8 | 145.9 | 0.0424 | 106.9 |
| ***Panel B: Ages 5 to 9*** |  |  |  |  |  | ***Panel B: Ages 5 to 9*** |  |  |  |  |
| Closure x 1(2020) | -0.964*** | -0.996*** | 3.168*** | -0.944*** |  | Closure x 1(2020) | -0.930*** | -0.958*** | 2.396*** | -0.881*** |
|  | (0.0552) | (0.0321) | (0.400) | (0.00845) |  |  | (0.0821) | (0.0134) | (0.398) | (0.0134) |
| Reopening x 1(2020) | -0.693*** | -0.358*** | -0.0182 | -0.282*** |  | Reopening x 1(2020) | -0.624*** | -0.276*** | -0.0879 | -0.181*** |
|  | (0.0519) | (0.0469) | (0.0456) | (0.0280) |  |  | (0.0396) | (0.0115) | (0.0680) | (0.0141) |
| County FE | X | X | X | X |  | County FE | X | X | X | X |
| Year FE | X | X | X | X |  | Year FE | X | X | X | X |
| N | 5772 | 5772 | 5687 | 5772 |  | N | 3900 | 3900 | 3879 | 3900 |
| Weekly Mean Pre-COVID | 69.86 | 326.1 | 0.0116 | 282.2 |  | Weekly Mean Pre-COVID | -0.774*** | -0.803*** | 1.391*** | -0.939*** |

Table S2 cont. The effect of dental office closures and reopenings on visit rates for rural vs. urban areas, Poisson regression, DID estimates and standard errors calculated using Shang et al. (2018)

| COUNTIES WITHOUT DENTAL SHORTAGE | | | | |  | COUNTIES WITH DENTAL SHORTAGE | | | | |
| --- | --- | --- | --- | --- | --- | --- | --- | --- | --- | --- |
| ***Panel C: Ages 10 to 14*** | **All-Cause ED** | **All Dental and Dental-Related** | **Proportion of Dental in ED** | **Dental Office** |  | ***Panel C: Ages 10 to 14*** | **All-Cause ED** | **All Dental and Dental-Related** | **Proportion of Dental in ED** | **Dental Office** |
| Closure x 1(2020) | -0.906*** | -0.859*** | 0.805*** | -0.843*** |  | Closure x 1(2020) | -0.941*** | -0.852*** | 1.076*** | -0.802*** |
|  | (0.0511) | (0.0171) | (0.262) | (0.00962) |  |  | (0.0777) | (0.0119) | (0.247) | (0.0173) |
| Reopening x 1(2020) | -0.524*** | -0.292*** | -0.252*** | -0.265*** |  | Reopening x 1(2020) | -0.481*** | -0.219*** | -0.260** | -0.167*** |
|  | (0.0479) | (0.0282) | (0.0941) | (0.0204) |  |  | (0.0192) | (0.00890) | (0.121) | (0.0136) |
| County FE | X | X | X | X |  | County FE | X | X | X | X |
| Year FE | X | X | X | X |  | Year FE | X | X | X | X |
| N | 5772 | 5772 | 5602 | 5772 |  | N | 3900 | 3900 | 3886 | 3900 |
| Weekly Mean Pre-COVID | 66.55 | 323.5 | 0.00578 | 297.2 |  | Weekly Mean Pre-COVID | 79.50 | 286.8 | 0.00748 | 268.1 |
| ***Panel D: Ages 15 to 19*** |  |  |  |  |  | ***Panel D: Ages 15 to 19*** |  |  |  |  |
| Closure x 1(2020) | -0.775*** | -0.808*** | 0.929*** | -0.822*** |  | Closure x 1(2020) | -0.715*** | -0.794*** | 1.410*** | -0.790*** |
|  | (0.0387) | (0.0113) | (0.201) | (0.0105) |  |  | (0.101) | (0.00621) | (0.211) | (0.00749) |
| Reopening x 1(2020) | -0.399*** | -0.222*** | -0.0672 | -0.225*** |  | Reopening x 1(2020) | -0.319*** | -0.156*** | -0.233 | -0.149*** |
|  | (0.0356) | (0.0205) | (0.0742) | (0.0199) |  |  | (0.0489) | (0.0106) | (0.148) | (0.0121) |
| County FE | X | X | X | X |  | County FE | X | X | X | X |
| Year FE | X | X | X | X |  | Year FE | X | X | X | X |
| N | 5772 | 5772 | 5707 | 5772 |  | N | 3900 | 3900 | 3882 | 3900 |
| Weekly Mean Pre-COVID | 94.45 | 278.3 | 0.0114 | 255.8 |  | Weekly Mean Pre-COVID | 115.4 | 258.9 | 0.0140 | 241.9 |
